# Editorial: Natural products in the treatment of Hyperuricemia, gout and other metabolic disorders

**DOI:** 10.3389/fphar.2023.1333257

**Published:** 2023-11-29

**Authors:** Ferid Abdulhafiz, Arifullah Mohammed, Kishore Kumar Chiruvella, Mallikarjuna Korivi, Mohd Farhan Hanif Reduan

**Affiliations:** ^1^ Faculty of Agro-Based Industry, Universiti Malaysia Kelantan, Kota Bharu, Malaysia; ^2^ Faculty of Veterinary Medicine, Universiti Malaysia Kelantan, Kota Bharu, Malaysia; ^3^ Department of Biochemistry and Biophysics, Lineberger Comprehensive Cancer Center, University of North Carolina at Chapel Hill, Chapel Hill, NC, United States; ^4^ Institute of Human Movement and Sports Engineering, College of Physical Education and Health Sciences, Zhejiang Normal University, Jinhua, China

**Keywords:** natural products, natural medicine, metabolic diseases, Hyperuricemia, gout

This Research Topic comprises research articles, systematic reviews, and meta-analyses. It emphasizes recent developments in natural products research and their pharmacological applications in treating hyperuricemia, gout, or metabolic disorders. This special Research Topic also explored the pharmacological efficacies of natural products, especially those used in cancer treatment. Scientific evidence has shown that natural products can enhance overall health, attenuate metabolic disorders and protect humans against a range of diseases. Hyperuricemia, characterized by an elevated level of uric acid in humans is closely associated with onset of gout. Approximately one-third of uric acid is produced endogenously, and the rest is generated during purine metabolism. In humans, around 70% of urate is excreted via the kidneys, and the remaining amount is excreted via the gastrointestinal tract. Elevated uric acid levels can lead to the deposition of uric acid crystals and acute inflammatory reactions in the joints. Various plant-derived substances have been shown to exhibit potent anti-hyperuricemia and anti-gout properties. For instance, *Baccharis trimera* (Less.) DC (Asteraceae) stem extracts showed a strong inhibitory property against xanthine oxidase activity, which is involved in uric acid production. The phytochemicals in *Baccharis trimera* stem, namely, trimera o-coumaric acid, palmitic acid, naringenin, protocatechoic acid, and linoleic acid may be contributed to these beneficial effects Song et al. Patients with gout typically represent with impaired wound healing mechanism, probably due to imbalanced inflammatory system or antioxidant homeostasis. A study showed that *Oroxylum indicum* (L.) Benth. Ex Kurz (Bignoniaceae) extracts treatment accelerated the wound healing process in rats, which might be due to the presence of various pharmacological phytochemicals Abdulhafiz et al. Since phytochemicals are known for their antioxidant and anti-inflammatory properties, *O. indicum* extracts also can be used to treat wounds or inflammation in gout Abdulhafiz et al.

Gout is a form of inflammatory arthritis that is resulted from persistent elevation of urate levels and formation of pro-inflammatory monosodium urate crystals in joints. In this context, supplementation of fermented jellyfish-collagen (*Rhopilema esculentum* Kishinouye, 1891) (Rhizostomatidae) to osteoarthritis obese rats reported to downregulate pro-inflammatory cytokines (tumor necrosis factor-α and cyclooxygenase-2), inhibit leptin and adiponectin production and improve antioxidant status Sudirman et al. Such anti-inflammatory and antioxidant activities of fermented jellyfish-collagen could be considered as a treatment for osteoarthritis or associated inflammatory diseases. Natural products are also widely used to treat several metabolic disorders, including diabetic nephropathy and various types of cancers. Chinese herbal formulation comprising of two or more bioactive compounds reported to be beneficial in reducing urinary albumin levels and preserving renal function in patients with diabetic kidney disease. Precisely, the Chinese herbal medicine can normalize the endoplasmic reticulum stress, and thereby manage the progression of diabetic nephropathy Wei et al. In line, a systematic review and meta-analysis evaluated the clinical efficacy and safety of the Niaoduqing granules, a Chinese traditional medicine composed of 16 herbs, in the treatment of diabetic kidney disease. This study concluded that adjuvant treatment with Niaoduqing effectively improved kidney function, inflammatory system, antioxidant status, lipid metabolism and glucose homeostasis in patients with diabetic kidney disease Song et al.

Although plant-based bioactive compounds are effective in treating various cancer types, the anti-cancer efficacy of herbal compounds is limited by poor solubility or bioavailability. In this regard, a study by Chittineedi et al. showed that polyherbal formulation conjugated into gold nanoparticles increased the solubility and anticancer efficacy. To be particular, treatment of breast cancer cells with polyherbal formulation conjugated gold nanoparticles represented by induction of cell cycle arrest, DNA damage, ferritin degradation and cell death (Chittineedi et al.. Furthermore, bioactive compound from *Nyctanthes arbortristis* L. (Oleaceae) also exhibits potent anti-cancer properties. *N. arbortristis* compounds and doxorubicin conjugated into gold nanoparticles triggered the production of intracellular reactive oxygen species, induced autophagic flux and early apoptosis in breast cancer cells Chittineedi et al. Our Research Topic highlighted that natural products are capable of treating various metabolic disorders, such as hyperuricemia, diabetic kidney disease and cancer.

